# Comparative effectiveness of antihypertensive drugs prescribed in Ethiopian healthcare practice: A pilot prospective, randomized, open label study

**DOI:** 10.1371/journal.pone.0203166

**Published:** 2018-09-11

**Authors:** Hayelom Gebrekirstos Mengesha, Abraha Hailu Welegerima, Abera Hadgu, Haftom Temesgen, Mala George Otieno, Kiflom Tsegay, Tedros Fisseha, Samuel Getachew, Zekarias Merha, Helen Tewodros, Jiksa Dabessa, Berhane Gebreegzabher, Pammla Petrucka

**Affiliations:** 1 Department of Pharmacy, College of Medicine and Health Science, Adigrat University, Mekelle, Ethiopia; 2 Departement of Internal Medicine, School of Medicine, Mekelle University, Mekelle, Ethiopia; 3 Departement of Pharmacology and Toxicology, School of Pharmacy, Mekelle University, Mekelle, Ethiopia; 4 School of Public Health, Mekelle University, Mekelle, Ethiopia; 5 College of Health Science, Department of Medical Biochemistry, Mekelle University, Mekelle, Ethiopia; 6 Adwa Hospital, Internal Medicine Unit, Adwa, Ethiopia; 7 Adigrat Hospital, Internal Medicine Unit, Adigrat, Ethiopia; 8 Kidst Mariam Hospital, Internal Medicine Unit, Axum, Ethiopa; 9 Mekelle Hospital, Internal Medicine Unit, Mekelle, Ethiopia; 10 Ayder Referral Hospital, Internal Medicine Unit, Mekelle, Ethiopia; 11 Department of Midwifery, Mekelle University, Mekelle, Ethiopia; 12 University of Saskatchewan, College of Nursing, Saskatchewan, Canada; 13 Adjunct Nelson Mandela African Institute of Science and Technology, Arusha, Tanzania; Cleveland Clinic Lerner Research Institute, UNITED STATES

## Abstract

**Background:**

Previous research has been highly suggestive that patients of African ancestry are less responsive to beta-blockers and angiotensin converting enzyme inhibitors. However, clinical practice within Ethiopia has continued to recommend all drugs for treatment of hypertension despite the lack of evidentiary support. Therefore this study aims to compare the effectiveness of the three major antihypertensive drugs currently prescribed in an Ethiopian health care setting to further the potential for evidence based prescribing practices.

**Methods:**

A prospective, randomized, open label comparative study was used to determine the mean reduction in blood pressure (primary outcome) and assess cardiovascular events (secondary outcomes) among patients receiving one or more of three common antihypertensive drugs (i.e., nifedipine, hydrochlorothiazide, and enalapril) in routine clinical practice between November 2016 and April 2017. Patients were followed for three months. Analysis was based on an intention-to-treat approach. One way analysis of covariance was used to compare the difference in therapeutic effectiveness in reducing blood pressure.

**Result:**

A total of 141 patients were randomized to one of three recipient groups—nifedipine (n = 47), enalapril (n = 47) or hydrochlorothiazide (n = 47). Three months after randomization, 44 patients in each group completed the follow-up. Patients randomized to nifedipine had significantly higher mean reduction in systolic blood pressure than those randomized to enalapril(p = 0.003) or hydrochlorothiazide(p = 0.036). The mean reduction in systolic blood pressure was -37.35(CI:-40, -34.2) in the nifedipine group; -30.3(CI: -33.5, -27.1) in patients receiving enalapril; and -32.1(CI:-35, -29.3) in patients assigned hydrochlorothiazide. However, nifedipine did not have a significance difference in reduction of mean diastolic blood pressure compared than those receiving enalapril (p = 0.57) or hydrochlorthiazide (p = 0.99).

**Conclusion:**

This study revealed that amongst the three drugs nifedipine was found to be the most effective drug in reduction of systolic blood pressure. Hydrochlorothiazide and enalapril did not show a difference in reduction of mean blood pressure. Further, long term randomized trials are highly recommended to inform revision of Ethiopia-centric hypertension treatment guidelines.

## Background

According to Kearney *et al*, 75% of deaths in Sub-Saharan Africa will be attributable to hypertension by 2020[1]. There is growing evidence that prevalence of hypertension is on the rise in most sub-Saharan Africa countries including Ethiopia [2,3]. A number of studies indicate that, in African patient populations compared with other population groups, hypertension in the former group is more severe, more resistant to treatment, and more likely to lead to immediate end organ damage and premature death [4–7]. Despite lifestyle adjustment and treatment options, the hypertension burden is increasing and globally, in adults, is expected to increase by 60% from 2000 to reach 1.56 billion afflicted in 2025 with majority in SSA [1]. A contributing factor for this trend is very low awareness about treatment and control among populations within low and middle-income countries (LMIC) including Ethiopia [8,9].

Data from outcomes studies and guidelines show that several classes of drugs, including angiotensin-converting enzyme inhibitors (ACE-I), angiotensin-receptor blocker, beta-blockers(BB), calcium channel blockers(CCBs) and thiazide-type diuretics, effectively lower blood pressure (BP) and reduce the complications of hypertension [5,10–14].

When treating hypertension, patients of African ancestry have typically responded better to CCBs and diuretics, while the response to BB and ACE-I is attenuated [5,15–17]. In a comparative efficacy study between black and white hypertensive patients in the United States of America, ACE-I based therapy was associated with poorer cardiovascular outcomes in hypertensive blacks [12] resulting in a recommendation by the 2014 Joint National Committee-8(JNC-8) for use of thiazide and CCB as first line therapy in African-Americans [13]. According to a recent systematic review, this attenuated effectiveness of BB and ACE-I may relate to low nitric oxide(NO) and high creatine kinase(CK) levels; however, other factors beyond pharmacokinetics were also associated such as genetic polymorphism, low renin levels, and a propensity towards ‘salt sensitivity’ amongst African-Americans as potential factors in the relatively poor response to ACE-I [12,16]. However, most conclusions were extrapolated from studies conducted in black people living in the United States and Europe, for which the outcomes may be affected by ethnic admixture and non-traditional lifestyles[18].

Ethiopian standard treatment guidelines intended for use in general hospitals and a new guideline released recently recommends use of thiazide diuretics, CCB, and/or ACE-I as treatment options for stage I and II hypertension without co-morbidities [10,11].

Due to lack of clinical evidence and contradictory findings [5,15–17], all three classes of antihypertensive drugs are being prescribed in Ethiopia. We hypothesized that the effect of these three antihypertensive drugs might be different in an Ethiopian population compared to other populations. To our knowledge, there is no evidence which addresses the comparative effectiveness of these classes of drugs in a routine clinical practice in African setting, in general, and Ethiopian context, in particular. Therefore, the aim of this study was to determine the comparative effectiveness of these three drugs in northern Ethiopia to establish evidence and insights to inform future effective treatment of hypertension and guideline development.

## Methods and materials

### Study area

This study was conducted in Tigray Region, northern Ethiopia, in its capital city (Mekelle) located 783 km from Addis Ababa, capital city of Ethiopia. Tigray Region is one of the 11 administrative states in Ethiopia with a total population of 5,055,999 (49.2% male and 50.8% female). The Ethiopian health system is structured into a three-tier system with the primary care level comprised of health posts, health centers and primary hospitals; the secondary care level of general hospitals; and the tertiary care level of specialized hospitals. In Tigray Region there are 15 public general hospitals, 1 specialized referral hospital, 20 primary hospitals, 204 health centers, and 712 health posts [19].

This study was conducted in 5 public hospitals (four of the 15 general hospitals and one of one specialized referral hospital), serving a catchment population of more than 6 million people. These hospitals have comparable infrastructure and capacity to diagnose and treat patients suspected of hypertension. Moreover, the chosen hospitals were convenient to follow up patients compared to other similar hospitals.

The general hospitals use Ethiopian standard treatment guidelines for general hospitals [10] for treating hypertension and its complication. The update of treatment options and guidelines depends on the update of treatment guidelines in accordance with the joint national committee recommendations and other European/American guidelines. In all hospitals, enalapril, nifedipine, and HCT are the most commonly prescribed medications for essential stage I and stage II hypertension from their respective classes. All the hospitals have inpatient and outpatient departments to manage cases of hypertension and its complications.

### Study design and period

A prospective, randomized, open label experimental study design was conducted from November 2016 to April 2017 in patients diagnosed with primary (essential) hypertension.

### Inclusion and exclusion

All patients diagnosed with stage I and stage II essential hypertension (**Table 1**), 18 years or older, and non-breast feeding, non-pregnant, and with no intention of pregnancy in the next three months met the inclusion criteria for this study. Patients experiencing a hypertensive emergency, having a known contraindicated to any of the study drugs (e.g., ACE-I to patients with angio-edema), secondary hypertension diagnosis, or a major co-morbidity (i.e., diabetes mellitus, kidney disease, heart failure, stroke, or coronary artery disease), or a body mass index greater than 40 kg/m^2^ were excluded.

**Table 1:** The stages of hypertension according to JNC- 7 [21].

Stage of hypertension	SBP	DBP
Normal	90–119	60–79
Pre-hypertension	120–139	80–89
Stage I	140–159	90–99
Stage II	≥160	≥100

DBP, diastolic blood pressure; SBP, systolic blood pressure

### Sample size and technique

To determine sample size, we considered previous studies conducted in African patients which would have similarity with our study and was supportive to detect the true effect. Therefore, we based our calculations on an average mean reduction in DBP from baseline for CCBs, ACE-I, and diuretics in mean (±SD) was -3.3 mm Hg (8), -3 mm Hg (7.2), and -2 mm Hg (7.6), respectively [14].

The formula for sample size determination was n = (Z_α/2_+Z_β_)^2^ *2*σ^2^ / d^2^, assuming the sample size is equally distributed across the groups, where Z_α/2_2 for a confidence level of 95%, α is 0.05 and the critical value is 1.96, Z_β_ for a power of 80%, β is 0.2 and the critical value is 0.84, σ^2^ is the population variance, which is assumed to be equal across the groups, and d is the difference we would like to detect which is 5 mmHg and assuming 10% for loss to follow up and withdrawal. First we calculated the sample size required for each drug by using the above formula and then we took the maximum sample size from the three calculated numbers and multiplied by three, yielding a required sample size of approximately 141. This was allocated to each group as 1:1:1.

### Participants, recruitment and randomization

Participants were newly diagnosed, uncomplicated, treatment naïve stage I or II essential hypertension, with DBP and SBP of above 90 mm Hg and 140 mmHg respectively. Patients recruitment and randomization was done by two different physicians. All hypertensive patients who appeared in the study hospitals during the study period and who fulfilled the inclusion criteria and gave written informed consent were recruited between November 2016 and February 2017 by a resident physician. After the patients were screened for eligibility, drugs were randomly assigned to each patient by a senior physician randomly. The method was simple randomization, using random generated numbers. When the patient appeared to the study hospitals the drugs were assigned to either of the three medications randomly. All study hospitals had their own centers and randomized patients in the same scenario. We did not make an attempt to alter the medications to make them identical and indistinguishable to the patients, partly because of feasibility and cost, as well as the aim of this study was to assess the effectiveness of these drugs in routine clinical practice. The drugs were prescribed to the patients randomly since the guidelines indicate that a patient with essential hypertension can take any of the three drugs [10,11].

### Outcome and treatment

The primary outcome (dependent variable) was mean reduction in BP as it is the best measure to predict cardiovascular outcomes [6]. It was calculated as the difference between BP value in each follow-up after starting therapy and baseline BP; thus, the average of the difference was the mean reduction in BP (SBP and/or DBP). Secondary outcomes considered included time to cardiovascular events, both mortality and morbidity, and time to achieving goal BP (i.e., normotensive or specified range). These secondary outcomes may include fatal and non-fatal myocardial infarction, heart failure, stroke, and coronary artery disease.

The independent variable considered was treatment type, representing three drugs (i.e., nifedipine, HCT, and enalapril) from the three classes. These included enalapril from the ACE-I, HCT from diuretics, and nifedipine from the CCB and were prescribed as follows: enalapril 5mg bid, HCT 12.5 mg po daily, and nifedipine 20 mg po bid. According to expert consultations and reviewing guidelines on treatment options, the dosages and frequencies for each drug were expected to have similar effects. However, if the BP was not managed and controlled with the initial doses and frequencies, the physicians were permitted to increase dose of the drug starting from the second period (i.e., first follow up) to the respective maximum doses (i.e., 40 mg nifedipine, 15 mg enalapril and 25 mg of HCT) being reached within two months of the follow up period. If the BP is still uncontrolled within two month period, the study physicians were mandated to change/shift treatment, initiate a combination therapy, and to consider the patient as lost to follow-up or cross-over.

### Data collection and follow up

One clinical pharmacist, one resident physician, and a senior physician, who work in each the study hospitals, were assigned for data collection and follow up. Recruitment and collecting baseline information was carried out by one resident in each study hospital when patients diagnosed with hypertension and appeared in the cardiology, inpatient internal medicine, and/or outpatient departments of the study hospitals. The clinical pharmacist worked with the physician during medication dispensing and following for adherence of the assigned medications. The senior physician randomizes the patients after they were recruited by the resident. Post-randomization started from day 7 of the first month follow up, the senior physician measured the BP, heart rate, adjusted dosages and/or medications according to need, identified any cardiovascular events, and added combination drug therapies based on the status of the patient.

A pilot study was conducted in Quiha Hospital to validate the questionnaire and its design. A face to face interview using a structured questionnaire developed based on previous studies was used to assess the socio-demographic characteristics, such as residence, gender, income, occupation, educational status, and marital status of participants at baseline.

Clinical characteristics, such as SBP/DBP, heart rate, and laboratory investigations (i.e., serum potassium, serum uric acid, serum cholesterol, urine tests for albumin, glucose, total cholesterol, low density lipoprotein, triglyceride, high density lipoprotein), electrocardiography, height and weight measurements were taken at baseline by the resident physician using an extraction form. Post randomization patients were followed for three month (Days 1, 7, 14, and 30 for the first month and once a month for the remaining two months) including assessment/measurement and reporting on a standardized form of SBP/DBP, any cardiovascular event, adverse event, side effects, and heart rate.

Mean of the SBP and DBP has previously been reported to be the best predictor of BP-related cardiovascular events [6]. Cardiovascular events and time to BP control were identified during the follow up period by the senior physician and recorded in the form of time to event.

All patients were followed until the end of the study regardless of participants’ outcomes. Patients who withdrew or a were lost to follow up from the treatment due to side effects, poor BP control (especially if the primarily prescribed drug is switched to other class), and other reasons were recorded and an explanation entered on the data collection form.

### Definition and assessment of variables

Blood pressure measurement including a sitting BP measured using mercury sphygmomanometers with appropriate cuff sizes according to the American Heart Association guidelines [20]. Clinical observers were encouraged to use the same BP monitor and the same observer for each patient visit and to use the non-dominant arm for all BP measurements throughout the study to optimize consistency in measurements. BP was measured after at least 5 minutes in the seated position. Three seated BP were measured at least 2 minutes apart, and reported a mean BP. The same procedure was followed throughout the follow up period for 3months.

Uncontrolled blood pressure was defined as average clinic SBP ≥140mmHg or DBP ≥90mmHg following the JNC-8 guidelines for patients less than 60 years old and >150/90 for patients >60 years old [13]. Dose escalation is defined as the increment in dose of the study drugs from the dose prescribed during randomization. Concomitant medication was defined as a medication (e.g., lipid lowering drugs) taken by a patient along with the randomized drugs, an approach assistive in assessing medication interactions. Co-morbidities was defined as a disease which was medically diagnosed (e.g., peptic ulcer disease) apart from hypertension and its complications.

Added medication (known as combination therapy) meant addition of a second of the three study drugs into the drug regime originally assigned during randomization (e.g., nifedipine added in to HCT). Medication switching was defined as shifting or changing the medication that was randomized in the beginning of the study to another alternative drug due to side effect or poor control of BP.

Adherence refers to the extent to which a patient follows the prescribed interval and dose regimen, which was assessed by pill count.

Height and weight were measured without shoes using a tape measure (to nearest 0.1 cm) and a validated digital scale (to nearest 0.1 kg) respectively in order to calculate body mass index status/change. Fasting blood specimens were collected from each participant after an overnight fast.

Stages of BP level was classified in to four stages and defined below (**Table 1**).

### Data management and analysis

Analysis was carried out based on intent to treat approach. To characterize the study population, descriptive statistics was performed using mean and standard deviation (SD) for continuous variables. For categorical variables, frequencies and percentages were calculated. Between-group comparison of continuous variables was performed using one-way analysis of variance. Categorical data were compared between groups using the χ2 (chi-square) test. We used analysis of covariance (ANCOVA) to model mean reductions in BP against the three drug classes. To account for patient differences, multivariable modeling using ANCOVA, considering covariates such as age, sex, baseline BP, and medication use, was built for both SBP and DBP. For all outcomes, P-values were adjusted for multiple tests using the Tukey HD method. All statistical tests were considered significant at a 2-sided P value of 0.05 and less and 95% CI was estimated. All analysis was carried out using SPSS^TM^ Version 20.0.

### Side effect assessment

Reported side effects or adverse events were assessed by the physician and clinical pharmacist with laboratory investigations conducted to affirm side effect relationship with the medications. Necessary interventions were rendered based on the severity and nature of the adverse effects and if serious and mandated a change in class of drug, the patient was considered as lost to follow up and was switched to the appropriate treatment.

### Ethical consideration

Ethical clearance was obtained from Mekelle University, College of Health Science, and Ethical Review Board. Written informed consent was obtained after of the purpose of the study. If they were not able to give written consent because of inability to read/write, consent with a witness was obtained. Anonymity and confidentiality of patients were maintained.

## Result

### Participants

**Fig 1:**
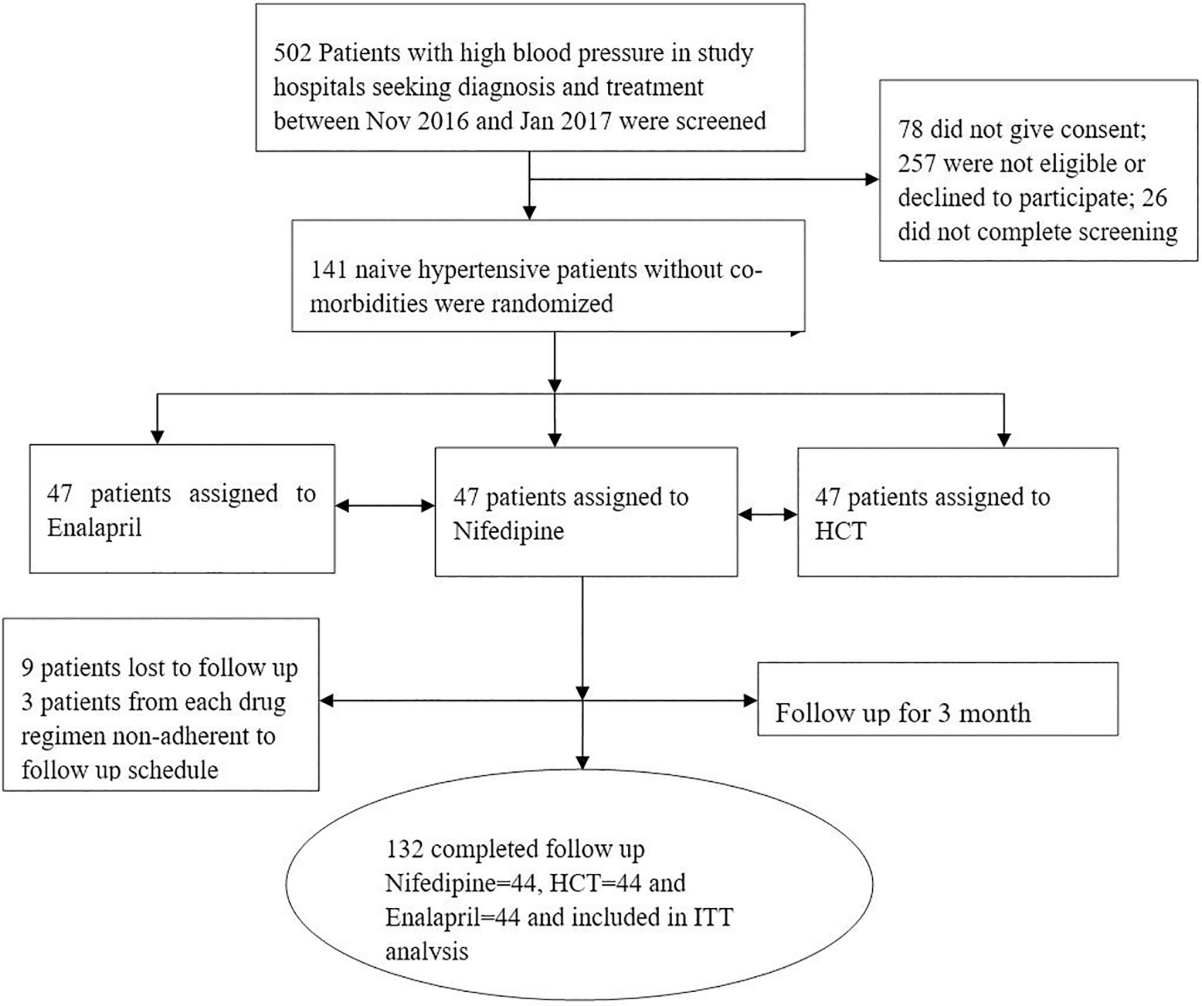
Randomization process of study participants.

From November 1, 2016, to January 31, 2017, a total of 502 patients were screened with 141 randomized across three treatment groups. Forty four patients in each treatment group completed the follow up and entered to analysis. There were nine lost to follow up, three patients in each group (i.e., nifedipine, enalapril and HCT) (**Fig 1**).

### Baseline characteristics of sociodemographic characteristics

Overall, the three groups were fairly balanced in all characteristics (**Table 2**). The majority of the participants were females, 85 (60.3%), unable to read and/or write (74 [52.2%]), and many were farmers (40.4%).

**Table 2:** Baseline characteristics of study (N = 141).

Characteristics	Total(N = 141)	Nifedipine(N = 44)	Enalapril(N = 44)	HCT(N = 44)	P-value
**Sex**					
Female (%)	85 (60.3)	28(33)	32(37.5)	25(29.5)	0.39
**Age**, (mean ± SD )	57(14.8)	59(14.7)	58(15.2)	54(14.3)	0.17
**Study site (%)**					
Adwa	36 (25.5)	12	12	12	
Ayder	18 (12.8)	6	6	6	
St. Mary	24 (17)	8	8	8	
Adigrat	51 (36.2)	17	17	17	
Mekelle	12 (8.5)	4	4	4	
**Education (%)**					0.25
Unable to read	74 (52.5)	27 (36)	28 (38)	19 (26)	
No Formal Education*	11 (7.8)	3 (28)	4 (36)	4 (36)	
1–8 grade	16 (11.3)	2 (12)	5 (31)	9 (56)	
9–12 grade	10 (7.1)	2 (20)	3 (33)	5 (47)	
College and above	28 (19.9)	12 (42)	6 (21)	10 (35)	
Marital status					0.71
Married	107 (75.9)	35 (32)	34 (32)	38 (36)	
Other	32 (22.7)	11 (34)	12 (37)	9 (29)	
**Residence(%), rural,**	87 (62.6)	17 (19)	18 (21)	17 (20)	0.81
**Occupation (%)**					0.32
Farmer	57 (40.4)	19 (34)	18 (31)	20 (35)	
Own business	17 (12.1)	2 (12)	7 (41)	8 (47)	
Housewife	27 (19.1)	10 (37)	12 (44)	5 (19)	
Government employee	30 (21.3)	12 (40)	8 (27)	10 (33)	
Other	8 (5.7)	3 (37)	1 (12)	4 (51)	
**Systolic BP**,(mm Hg) mean(SD)	166 (17.2)	172.9 (18.5)	162.7 (13.8)	163.9 (17.7)	0.007
**Diastolic BP**, (mm Hg) mean(SD)	96 (10.8)	97.9 (11.5)	94.8 (11.4)	92.1 (9.25)	0.34
**Cholesterol,** mg/dl mean(SD)	174.1 (32)	181.2 (30.6)	166.6 (37.3)	174.6 (27.6)	0.33
**Heart Rate,** bpm, mean(SD)	82.4 (10.7)	81.5 (9.4)	82.5 (11.9)	83.3 (10.9)	0.76
**BMI** (kg/m^2^), mean(SD)	22.5 (3.9)	23 (4.3)	22.1 (3.9)	22.44 (3.3)	0.72
**FBS**, mg/dl, mean(SD)	100 (12)	99 (13)	104 (10)	98 (11)	0.43
**Creatinine**, mg/dl, mean(SD)	0.8 (0.25)	0.78 (0.23)	0.76 (0.18)	0.85 (0.32)	0.29

*indicates no formal education; P-value based on one way ANOVA for continuous variables and chi-square for categorical variables;BP,blood pressure;BMI,body mass index;FBS, Fasting blood sugar

The overall mean (SD) age of patients was 56.7(14.8) the mean of baseline SBP was 166.5(17.2) and there was a significant difference in distribution between the study drugs (P = 0.007); whereas the mean DBP was 96(10.8) with a similar distribution across the groups (P = 0.34). The mean (SD) baseline heart rate was 82.4(10.7) with no difference in distribution (p = 0.76).

### Assessment of patients at the end of the follow up

Regarding time to BP control, with patients assigned to HCT showed the fastest BP control in 2.5(2.3) weeks followed by nifedipine 3.26(3.2) weeks and, finally, enalapril 3.7(3.8) weeks, there was no significant difference between the study drugs (P = 0.22). At the end of the study, mean heart rate was reduced to 75.9(9.4), 78.02(11.1) and 78.5(8.8) bpm in the nifedipine, enalapril and HCT groups yielding no significant difference in the study arms (p = 0.42). Few patients 4 (3%) reported side effects like dry cough, headache, and nasal stiffness. By the end of the follow up BP was controlled in the majority of patients 122(91.7%). Dose escalation occurred in nearly one third [44(32.5%)] of patients (enalapril was from 5 mg increased to 10 mg, nifedipine from 20 to 40mg and HCT 12.5 to 25mg). Only one cardiovascular event occurred in a single case of heart failure (**Table 3**). After two months follow-up nifedipine was added to four hypertensive patients randomized to HCT initially and to two patients assigned to enalapril, while enalapril was added to two patients randomized to nifedipine; and HCT was added to one patient commenced on nifedipine. There was one treatment shift in a patient randomized to ACE-I due to adverse effect of dry cough.

**Table 3:** Outcome of the follow up of study subjects (N = 141).

Characteristics	Total(N = 141)	Nifedipine(N = 44)	Enalapril(N = 44)	HCT(N = 44)	P-value
**Concomitant medication**					0.33
Yes (%)	9 (60.3)	1(19)	4(45)	4(45)	
**Dose Escalation (%)**					0.81
No	91(64.5)	31(34)	35(38)	25(28)	
Yes	44(31.3)	12(27)	10(23)	22(55)	
Missing	6(4.2)				
**Cardiovascular event (%)**					0.34
Yes	1(0.7)			1	
No	140(99.3)	47	47	46	
**Side Effects (%)**					0.43
No	137(97)	45	46	46	
Yes	4(3)	2	1	1	
**Additional Medication (%)**					0.37
No	120(85.1)	40(33)	41(33.5)	39(32.5)	
Yes	9(6.4)	2(23)	2(23)	5(54)	
Missing	12(8.5)				
BP* **control (%)**					0.46
Yes	122(87.9)	38(31)	43(35)	41(34)	
No	10(7.8)	4(40)	2(20)	4(40)	
**LSTFUP+(%)**	9(6.4)				0.18
**Co-morbidities (%)**					
No	93(65.9)	34(36)	26(28)	33(36)	
Yes	46(32.7)	13(28)	20(44)	13(28)	
Missing	2(1.4)				
**BP Control in weeks, mean (SD)**	3.1(3.2)	3.26(3.2)	3.7(3.8)	2.5(2.3)	0.27
HR end, bpm**, mean (SD)	77.5(9.5)	75.9(9.4)	78.0(11.1)	78.5(8.8)	0.42
Systolic BP* **at 7 weeks, (**mm Hg) mean(SD)	144(18)	141.8(20.5)	145.8(14.9)	145.1(18.7)	0.53
**Systolic BP at 14 weeks**, mean(SD)	137(16.4)	135.4(18.7)	138.3(15.6)	138(14.8)	0.66
**Systolic BP at 1 month**, mean(SD)	130(15.2)	128.1(16.2)	131.1(14.8)	131.1(15)	0.58
**Systolic BP at 2 months,** mean(SD)	127.2(13.8)	125.7(11.3)	130.4(17.1)	125.5(11.7)	0.16
**Systolic BP at 3 months**, mean(SD)	127.3(15)	126.4(14.7)	129.4(15.1)	126(15.1)	0.49

P-value based on one way ANOVA for continuous variables and chi-square for categorical variables

+means lost to follow-up

*, blood pressure

**bpm, beats per minute

### Mean reduction in BP

#### Effectiveness of the three study drugs in reducing SBP

On bivariable analysis using ANCOVA of all socio-demographic and clinical variables, baseline SBP (P = <0.001) and DBP (P = 0.007) was significantly associated with mean reduction in BP. Other variables did not have significant association.

On preliminary checks to ensure that there was no violation of the assumptions of normality, linearity, homogeneity of variances, and reliable measurement of the covariate, there was no violation in all parameters. Interaction was also checked between the independent variables and outcome variable in the study drugs by including an interaction term in the ANCOVA model, which yielded no significant interaction.

After adjusting for age and baseline SBP, there was a significant difference between the three drug groups in mean reduction of SBP, F = 4.89, P = 0.009, partial eta squared = .072 which indicates a moderate effect size according to Chen’s 1988 estimation [22]. There was a strong relationship between the baseline systolic hypertension on the mean reduction in SBP test after adjusted for drug groups, as indicated by a partial eta squared value of 0.52 which means 52% of variance in mean reduction of BP was explained by baseline SBP (**Table 4**).

**Table 4:** ANCOVA model test and effectiveness of drug types after adjusted for baseline BP and age.

Source	Sum of squares	DF	Mean square	F-value	P-value	Partial ETA square
Model	18300	4	4575	41.6	<0.001	0.56
Intercept	6126.9	1	6126.9	55.7	<0.001	0.35
Baseline SBP	14059.3	1	14059.3	127.9	<0.001	0.52
Age	0.7	1	0.7	0.06	0.93	0
Drug type	1076.5	2	538.3	4.89	0.009	0.072
Error	13963.8	127	109.9			

#### Effect in reduction of mean SBP and multiple comparisons

Adjusted for baseline SBP and age those randomized nifedipine, had significantly higher mean reduction in SBP. The mean reduction in SBP was -37.35(CI:-40, -34.2) in groups assigned to nifidipine, -30.3(CI:-33.5, -27.1) in patients assigned to enalapril and -32.1(CI:-35.-29.3) in patients assigned HCT. The mean SBP reduction was -7.03 mmHg(CI:-11.6,-2.49) higher [which is mean in those randomized to nifedipine—Mean in those randomized to enalapril] in those randomized to nifedipine than those randomized to enalapril(p<0·003).

The mean difference of in reduction of SBP in the nifedipine groupwas -4.88(CI:-9.43,-0.33) higher than those assigned to HCT. There was a significance difference in reducing SBP between nifedipine and HCT (p = 0.036). But there was no significant difference between HCT and enalapril (P = 0.34) (**Table 5**).

**Table 5:** Adjusted mean reduction in SBP and mean difference between the study groups.

Drug type	Comparator	Mean difference*	Mean(CI) reduction SBP	P-value	95%CI
Nifedipine	Enalapril	-7.03	-37.35 (-40,-34.2)	0.003	-11.6,-2.49
	HCT	-4.88		0.036	-9.43,-0.33
Enalapril	Nifedipine	7.03	-30.3 (-33.5,-27.1)	0.003	2.4,11.5
	HCT	2.15		0.34	-2.3, 6.6
HCT	Enalapril	-2.15	-32.5 (-35,-29.3)	0.34	-6.6,2.3
	Nifedipine	4.88		0.036	0.33,9.43

HCT, hydrochlorothiazide; CI, confidence interval; SBP, systolic blood pressure

* shows difference between the drug type and the comparator

#### Effect of drugs in reducing mean DBP

After adjusting for age and baseline DBP, there was no a significant difference between the three drug groups in mean reduction of DBP, F = 4.7, P = 0.8, partial eta squared = 0.003 which indicates a small effect size according to Chen’s 1988 estimation [22]. There was a strong relationship between the baseline DBP hypertension and mean reduction in DBP after adjustment for the independent variable drug types, as indicated by a partial eta squared value of 0.56 which means 56% of variance in means reduction of BP was explained by baseline DBP (**Table 6**).

**Table 6:** ANCOVA model test and effectiveness of drug types after adjusted for baseline DBP and age.

Source	Sum of squares	DF	Mean square	F-value	P-value	Partial ETA square
Model	7841.1	4	1960.3	42.32	<0.001	0.56
Intercept	4166.5	1	4166.5	89.9	<0.001	0.41
Baseline DBP	7626.2	1	7626.2	164.7	<0.001	0.56
Age	216.8	1	216.8	4.7	0.03	0.036
Drug type	19.67	2	9.83	0.21	0.8	0.003
Error	5881.4	127	46.31			

#### Mean difference between the study drugs in reduction of DBP

The mean reduction in DBP was -15.66(CI:-17.6, -13.6) in groups assigned to nifedipine, -14.8(CI:-16.8, -12.75) in patients assigned to enalapril and -15.62(CI:-17.6, -13.5) in patients assigned HCT. The mean difference between nifedipine and enalapril was -0.83(CI:-3.7,-2.08) while it is -0.014 between nifedipine and HCT (CI:-2.9, 2.89). There was no a significant difference in reducing DBP between nifedipine and HCT (P = 0.99), nifedipine and enalapril(P = 0.57) (**Table 7**).

**Table 7:** Adjusted mean reduction in DBP and mean difference between the study groups.

Drug type	Comparator	Mean difference*	Mean(CI) reduction DBP	P-value	95%CI
Nifedipine	Enalapril	-0.83	-15.66 (-17.6,-13.6)	0.57	-3.7,2.0
	HCT	-0.014		0.99	-2.9,2.8
Enalapril	Nifedipine	0.83	-14.8 (-16.8,-12.75)	0.57	-2.05,3.7
	HCT	0.81		0.57	-2.08,3.71
HCT	nifedipine	0.014	-15.62 (-17.5,-13.5)	0.99	-2.89,2.9
	enalapril	-0.81		0.57	-3.7,2.08

*shows the difference in mean between the drug type and comparator.

## Discussion

In this study, we hypothesized that the effect of enalapril, nifedipine and HCT on reduction of BP are different. We found that nifedipine was superior in mean reduction of SBP (P = 0.007) but with respect to DBP no significant difference in effect was evidenced (P = 0.57).

No published evidence compared the effects of first line antihypertensive drugs in Ethiopia, resulting in reliance on evidentiary extrapolation from other studies and populations. There are few trials which were conducted in an African context as evidenced in a recent meta-analysis [16]. Guidelines in Ethiopia recommended the use of thiazide diuretics as first line therapy, but it also considers CCB or ACEI as first line therapy as well [10,11]. Evidence-based European and American guidelines show CCB and thiazide diuretics as first line therapy and ACE-I as second line therapy [12,13]. A systematic review in Africa found a descending order of effectiveness as CCB followed by thiazide diuretics and then ACEI [16].

The findings in this study align with the guidelines on the effect of CCB in SBP but it contradicts the recommendations respecting HCT and enalapril in Ethiopia and Africa [11–13,16,23]. In this study, we did not evaluate the cost effectiveness of the drugs, a deficit that could affect the development of guidelines thereby necessitating further investigations.

Variation in reducing BP could be due to genetic variation of study participants compared to African Americans and other sub-Saharan Africans or due to environmental factors. The study findings suggest that nifedipine was most effective in reducing SBP while enalapril had a similar effectiveness profile to HCT, which contrasts with previous guidelines [11,13] and findings that nifedipine had similar efficacy with HCT, and enalapril had the lowest efficacy of this trio of drugs [16]. Regarding mean reduction in DBP, there was no significant difference. Although the study showed relative superiority of nifedipine in magnitude of mmHg, this study evaluated the effectiveness using an outcome of mean reduction in BP which might not be the ultimate endpoint to assess the effectiveness of antihypertensive drugs. There is strong evidence that mean reduction in pressure is the best measure to predict future cardiovascular-related morbidity and mortality [6] and it is recently revealed that intensive BP control is more effective in reducing cardiovascular outcomes compared to the traditional way of controlling BP to 140/90 mmHg [24].

### Effectiveness and possible mechanism of action

CCB, particularly nifedipine, were effective to decrease BP in this study population. Nifedipine is metabolized extensively by CYP 450 enzymes, specifically CYP 3A4 enzyme [25]. Genetic polymorphism in this enzyme to metabolize CCBs (like amlodipine) has been previously reported in individuals of African ancestry and it has been shown that different alleles of the same gene of CYP 3A4 had increased response to the control of mean arterial pressure [26].This could be a possible explanation for the effectiveness of nifedipine in our study population compared to other populations. Moreover, nifedipine is reported to have a lower clearance rate in African patients [27]. Additionally, research has suggested that African patients have high creatine kinase with enhanced smooth muscle contractility and low NO level [28–30]. Furthermore, CCB have been found to be effective in salt sensitive populations in contrast to ACEI [31]. Therefore the above peculiar mechanisms of action of CCB in people of African ancestry and previous evidence strengthen our finding that nifedipine is the optimal drug in our study populations.

In contrast to the current practice, our study population had lower response to HCT when compared with nifedipine and similar response when compared with enalapril. This remained valid on subgroup analysis by stage I and II hypertension. Thiazide diuretics are evidenced as the most effective drugs to reduce BP in African patients; hence, they are widely prescribed in routine clinical practice. This pattern is attributed to persons of African ancestry reported to have a tendency to retain salt [7,32], which is linked to a primary renal mechanism, as studies confirmed that increased sodium retention does not appear to be secondary to increased production of aldosterone, deoxycorticosterone, cortisol, or 18-hydroxycortisol [33]. However, this was not reflected in our study population.

The relatively lower effect of HCT compared to nifedipine could be related to genetic polymorphism as reported before [16] and, recently, a genome wide and gene based meta-analysis identified 3 novel loci (regulatory regions gap junction protein α1 gene (GJA1) and forkhead box A1 gene (FOXA1), relevant for cardiovascular and kidney function and β- and steroid δ-isomerase 1 gene3β-hydroxysteroid dehydrogenase enzyme and plays a crucial role in the biosynthesis of aldosterone and endogenous ouabain influencing BP response to HCT [34]. Moreover, high levels of creatine kinase show a higher tendency to retain salt [32]. It is not clear why our study populations were less responsive and one might postulate a moderate tendency to retain salt or moderate level of circulating rennin in Ethiopian patients although another study showed rennin is supposedly lower in other Africans [35]. Therefore, this variance calls for further investigation to explore the reason(s) associated with a relatively lower response of HCT in an Ethiopian population.

As was hypothesized, enalapril was not relatively effective in reducing BP compared to nifedipine. Previously it was predicted that a low response of ACEI is due to low rennin level and high salt retention tendencies in African people [35]; although evidence is inconsistent on the attenuated effect of ACEI whether it is due to low rennin or not [31,36]. Although lower salt level improves the effectiveness of ACE-I response to BP reduction, it is likely not the only reason [16] fully explaining the gap evidenced between African and European ancestral groups [37].

Inhibition of ACE-Is not the only mechanism that works but also ACEI promote NO synthesis in the endothelium [38]. Studies show that ACE-I does not only act by inhibiting angiotensin converting enzyme but may also act by activating production of NO [28–30]. A lower level of NO in African ancestry was reported which could contribute to the attenuated effect of ACE-I in African ancestry [16].

Nevertheless, it is unclear why HCT and nifedipine were not statistically different from enalapril in reduction of mean DBP and with HCT in decreasing SBP. This clearly indicates a need for further molecular and human studies.

### Mean reduction in SBP and DBP

In this study, the mean reduction in both in SBP& DBP was generally higher when compared to previous studies [14,16,31,39,40]. The reason for this pattern could be because of the higher baseline BP recorded in some patients. However, this deviation was adjusted in the ANCOVA analysis model.

The mean reduction in SBP in the nifedipine group was higher compared to previous studies [16,31]; although lower compared to an earlier study which had included patients with BP of up to230/110[41] which found a SBP reduction of -58.5mmHg compared to our study -37 mmHg. Collectively, the effect of CCB in terms of mean reduction in SBP ranges from16.9mmHg [16] up to 21.15mmHg [14]. Regardless, nifedipine in our study was the optimal drug to reduce BP both in terms of measured change and in comparison to the other study drugs. This result was similar in DBP compared to previous studies [14,16].

Diuretics were also as good as CCB in reduction of BP compared to other studies [16]. Although the difference is not significant, the -32.5(-35,-29.3) and -15.62(-17.6, -13.5) reduction in SBP and DBP respectively is higher compared to a previous study [42] which found SBP mean (SD) to reduce by 12.1(9.6) while the mean (SD) DBP reduced by 11(7.3). In general, the pooled effect of diuretics in reduction of mean SBP and DBP reflected 15.2mmHg (10.95) and 2mmHg (7.6) reduction from baseline respectively [14]. Our results were higher for both SBP and DBP compared to an African-centric meta-analysis conducted which showed an average reduction in SBP and DBP for diuretics of 15(CI: 13.1–17) and 10.7(CI: 9.5–11.9) respectively. Inclusion of patients with relatively high BP might contributed to the greater reduction in BP, leading to future investigation potentials on whether this reduction is associated with greater decrement in cardiovascular related mortality and morbidity.

In our investigation, the mean reduction in SBP and DBP by enalapril was -30.3(-33.5,-27.1) and -14.8(-16.8, -12.75) respectively. This is higher than a previous trial which compared effect of enalapril against control group resulting in a reduction of SBP by -7.1(13.2) versus 0.0(13.2) and DBP of -6.2(8.0) versus 0.0(8.0) [31]. In general the pooled effect of ACE-I in mean reduction of SBP and DBP found a -8.4(14) and -3 mmHg (7.2) reduction from baseline, respectively [14]. Moreover, our findings are also higher than results from a meta-analysis conducted recently in Africa which showed an average reduction in SBP and DBP for ACE-I of-8.5(CI: -7,-9.9) and -8(CI: -7.1,-8.9) respectively. Therefore, this clearly showed that ACE-I/enalapril has a superior efficacy in our study population compared to other African ancestors. This finding indicates the need for further molecular investigation to identify the reason whether it is because of genetic polymormphism, moderate renin levels, or creatine kinase and moderate NO level compared to other African patients.

### Study limitations and strengths

This study was an initial comparative effectiveness research with prospectively collected data and follow- up of patients established a baseline of reliable and valid data. Randomization, multi-site, and multi-practitioner involvements strengthened the reduction of bias. Furthermore, the result of randomization indicated distribution of baseline characteristics were almost balanced across the study groups which indicates the success of randomization. However, there was a difference in baseline SBP which may overestimate the effect of nifedipine.

The major weaknesses of this study were small sample size and short period of follow up, limiting the determination of cardiovascular events as a direct indicator for effectiveness antihypertensive drugs. Nevertheless, mean reduction in BP is a good predictor of cardiovascular event. In addition, the study was not blinded and concealment of medication allocation was not complete, which may impact on estimation of effect size.

## Conclusions

This study revealed that nifedipine was superior in reduction of BP in our target population of newly diagnosed Ethiopian primary hypertensive patients. This finding showed significance in reduction of SBP compared to HCT and enalapril. In reduction of DBP there was no significant difference but nifedipine was the topmost in decreasing the magnitude of mmHgs. In contrast to previous guidelines and recommendations, HCT and enalapril had similar effect in reduction of BP through this study. This finding warrants a multi-year, large sample size, longitudinal or experimental study to enhance evidence informed Ethiopian hypertension treatment guidelines.
